# Sorption Hysteresis: A Statistical Thermodynamic Fluctuation Theory

**DOI:** 10.1021/acs.langmuir.4c00606

**Published:** 2024-05-23

**Authors:** Seishi Shimizu, Nobuyuki Matubayasi

**Affiliations:** † York Structural Biology Laboratory, Department of Chemistry, University of York, Heslington, York YO10 5DD, United Kingdom; ‡ Division of Chemical Engineering, Graduate School of Engineering Science, Osaka University, Toyonaka, Osaka 560-8531, Japan

## Abstract

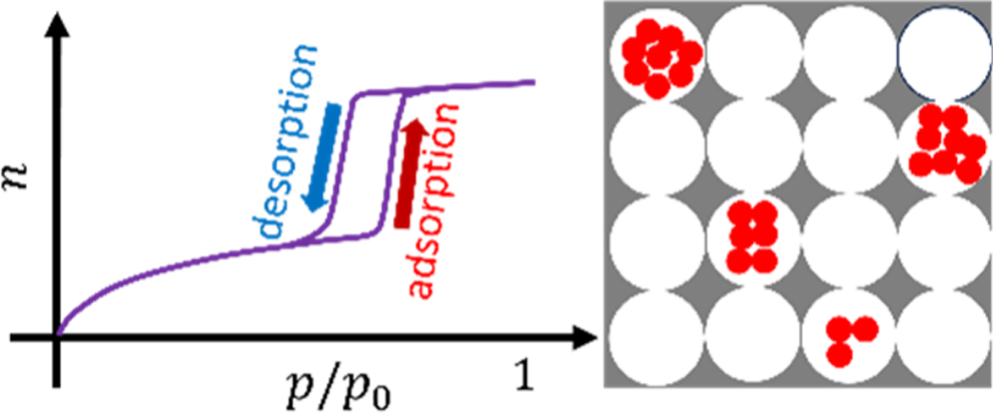

Hysteresis is observed commonly in sorption isotherms of porous materials. Still, there has so far been no unified approach that can both model hysteresis and assess its underlying energetics. Standard approaches, such as capillary condensation and isotherms based on interfacial equations of state, have not proved to be up to the task. Here, we show that a statistical thermodynamic approach can achieve the following needs simultaneously: (i) showing why adsorption and desorption transitions may be sharp yet continuous; (ii) providing a simple (analytic) isotherm equation for hysteresis branches; (iii) clarifying the energetics underlying sorption hysteresis; and (iv) providing macroscopic and nanoscopic perspectives to understanding hysteresis. This approach identifies the two pairs of parameters (determinable by fitting experimental data) that are required to describe the hysteresis: the free energy per molecule within the pore clusters and the cluster size in the pores. The present paper focuses on providing mechanistic insights to IUPAC hysteresis types H1, H2(a), and H2(b) and can also be applied to the isotherm types IV and V.

## Introduction

**Figure 1:**
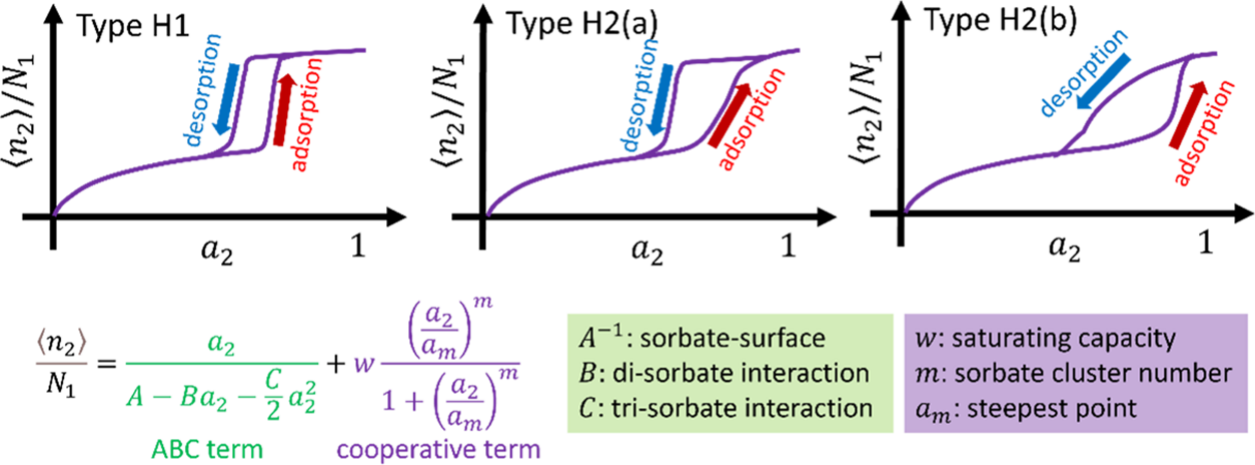
(Top). The IUPAC hysteresis types that this paper focuses on. Type H1 hysteresis loop “is found in materials which exhibit a narrow range of uniform mesopores”.^4^ Type H2 is found in “more complex pore structures in which network effects are important”.^4^ The steeper desorption branch of type H2(a) is attributed to “pore blocking/percolation in a narrow range of pore necks or to cavitation-induced evaporation”.^4^ Type H2(b) is “typical for materials with a narrow pore cavity size distribution and in the absence of percolation effects,”^63^ whose shallow desorption branch is “also associated with pore blocking, but the size distribution of neck widths is now much larger.”^4^ (Bottom) The isotherm equation derived in this paper (eq 16a) with a brief summary of the physical interpretations of its parameters.

Hysteresis in sorption isotherms (i.e., the existence of the adsorption and desorption branches)^1−3^ is observed frequently in the vapor (gas) isotherms of porous materials.^3,4^ The shape of a hysteresis loop is known to be “fairly closely related” to the structure and network of the pores and the underlying adsorption mechanism (Figure 1).^4^ However, there is still a gap between the proposed mechanistic insights (Figure 1) and the isotherm equations in an analytically tractable form. To fill this gap, the following aims of the present paper will be the key:

I. to show why adsorption and desorption transitions may be sharp yet continuous;
II. to derive a simple (analytic) isotherm equation for hysteresis branches;
III. to clarify the energetics underlying sorption hysteresis; and
IV. to offer macroscopic and nanoscopic perspectives to understanding hysteresis.

Our theoretical foundation is the statistical thermodynamic fluctuation theory^5−9^ based on sorbate number correlations and the interfacial Kirkwood–Buff integrals.^8,10^ Their link to molecular distribution functions shares the language not only with atomistic simulations^11−13^ that have been successful in reproducing sorption hysteresis but also with liquid solutions,^14−19^ colloids and nanoparticles,^20−22^ and interfaces.^5,6,8,9,23^ With the language of molecular distribution, our four aims will provide a link between the collective behavior of sorbates to the energetics of adsorption and desorption via an analytically tractable theory.

In the following, we will identify the reasons why these four aims have been difficult to achieve simultaneously by the conventional approaches to analytic isotherms (i.e., capillary condensation and isotherm models) and why the statistical thermodynamic theory of sorption^7,24^ is capable of overcoming their limitations.

### Capillary Condensation Models

Why is a higher relative pressure required for the adsorption transition (i.e., the sudden rise of isotherm) than the desorption transition (i.e., the sudden drop)? Capillary condensation^1−4,25,26^ answers this question based on the following assumptions: (i) vapor–liquid equilibrium takes place within a pore; (ii) a sudden rise/drop of an isotherm branch is analogous to the vaporization/condensation of the bulk liquid; and (iii) the critical pressure for vaporization/condensation in (ii) depends on the pore size.^1−4,25,26^ Consequently, the lower critical pressure for the desorption line comes from the smaller space within the pore available for vapor as has been shown via the Kelvin equation and its modifications.^4,25,26^ This is the foundation for the classical approaches to determining pore size distributions, such as the Barrett–Joyner–Halenda (BJH)^27^ and t-method.^28^ However, the Kelvin equation significantly underestimates the pore sizes of uniform mesopores (such as MCM-41),^4,29^ which contributes to inaccuracies in the isotherm equations on the nanoscale (aims II and IV). Moreover, the macroscopic thermodynamic nature of the Kelvin equation makes it difficult to explain why the transitions are sharp yet continuous for micropores and mesopores (aim I).

### Isotherm Models

Some isotherm models have been successful in reproducing hysteresis, including (i) the site-specific adsorption models, with the introduction of lateral sorbate–sorbate interactions, such as the Frumkin^30^ and Fowler–Guggenheim models,^31−33^ and (ii) the models based on the equations of states (EOS) for the spreading pressure,^1,33,34^ such as Hill-de Boer.^35,36^ Hystereses in these models arise from the multivalued nature of sorbate activity (when expressed as a function of the amount of sorption), analogous to first-order phase transitions.^37^ However, these models introduce transition discontinuity *a priori* rather than explaining why transitions are sharp yet continuous (aim I). In addition, the isotherm equations are often implicit functions,^33^ which makes it difficult for fitting experimental data (aim II).

### Statistical Thermodynamic Quasi-equilibrium Isotherms

We recently developed a universal approach to sorption isotherms^5,6,8,38^ through (i) a generalization of the statistical thermodynamic fluctuation theory for solutions^14−18^ to interfaces, in combination with (ii) the Gibbs isotherm to arbitrary interfacial geometry.^5^ This new theory provides a common language for experimental isotherms and atomistic simulations (such as molecular dynamics simulations^11−13^ and numerical density functional theory^39−42^) via molecular distribution functions, sorbate number correlations, and the Kirkwood–Buff integrals^8,10^ as a natural extension of the theory for liquid solutions,^14−19^ colloids and nanoparticles,^20−22^ and interfaces.^5,6,8,9,23^ Our initial approach to hysteresis branches followed the traditional assuption^43−46^ that thermodynamic isotherms can be utilized for long-lived metastable states, such as adsorption and desorption branches.^7^ By circumventing the difficulty of explicitly treating hysteresis branches,^7,9,24^ our cooperative isotherm was derived directly from the excess number relationship (i.e., the fundamental relationship of the fluctuation sorption theory) and was applied successfully to adsorption on porous materials.^7,24^ This approach is capable of (i) expressing isotherm in an analytic form (aim II) and (ii) attributing a large (yet not infinite) isotherm gradient to sorbate cluster size (aim I).^7,24^ However, how the distinct energetics of adsorption and desorption can be described (aim III) has remained unclear.

### Our Approach

To achieve all four aims for the elucidation of sorption hysteresis, we will take the following four-step strategy.

#### Objective I: To Understand Why Transitions Are Sharp yet Continuous^3,4^

To fulfill this objective, we will derive (in the Theory section) the thermodynamic stability conditions for the pore (nanoscale) and the entire interface (macroscale) for which sorbate number fluctuation will play a key role. This will be achieved by extending our recent thermodynamic stability theory under nanoscale confinement^47^ to the vapor/solid interface. From this approach, the isotherm branch steepness, which has played an important role in hysteresis classification (Figure 1), will be translated to microscopic insights.

#### Objective II: To Derive the Branch Isotherm Equations

Based on the sorbate number fluctuation underpinning the stability condition, we will derive an analytical isotherm equation. Its parameters, whose interpretive clarity comes from the fluctuation theory, will capture the mechanism underlying the hysteresis types.

#### Objective III: To Reveal the Energetics Underlying Hysteresis Branches

Linking an isotherm branch to the interfacial free energy (from the Gibbs isotherm) is essential for understanding the energetic basis of hysteresis, as will be achieved in the Theory section. We will show how (i) the stabilization of sorbate at the interface and (ii) the change of sorbate cluster affect the interfacial free energy (see the Results and Discussion section). Through this, we will link the mechanistic insights in the literature (e.g., “delayed condensation” and “pore blocking/percolation”^4^) to the interfacial free energy.

#### Objective IV

An alternative to the fluctuation theory to achieve objectives I–III comes from Hill’s thermodynamics of small systems^48−50^ via a consideration of vapor–liquid transition within a pore and the entropy of arranging vapor and liquid pores throughout the interface. Its equivalence to the fluctuation theory will be demonstrated when there is no correlation between the pores (see the Results and Discussion section).

## Theory

This section furnishes the theoretical foundation necessary for our four objectives set out in the Introduction section, in preparation for achieving them in the Results and Discussion section.

### Quasi-thermodynamic Stability Theory

#### Macroscopic Formalism

To understand why the transitions are sharp yet continuous (objective I of the Introduction section), we will develop a thermodynamic stability theory for porous adsorption. To establish the stability theory, here, we generalize our recent work on confined solutions^47^ to interfaces. We consider a vapor–solid system with an interface, together with the two reference systems, i.e., the bulk gas (vapor) (*g*) and solid (*s*) systems with no interface. The interfacial free energy, following Gibbs,^51^ is defined as the difference between that of the system and those of the reference systems, generalized in our previous papers to interfaces of arbitrary geometry and porosity.^5^ Absorption and sorbent structure changes can also be considered by our theory. The three systems are surrounded by the reservoir. The key quantity is the minimum excess work done by an external medium on the system + reservoir, δ*R*, that accompanies the exchange of sorbates (species 2), which can be expressed (see Supporting Information section A) in the following quadratic form for the system and the reference states:
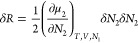
1where *N*_1_ and *N*_2_ are the numbers of sorbent and sorbate, δ*N*_2_ is the deviation from the mean, and μ_2_ is the chemical potential of the sorbate. Our goal is to obtain an expression for the minimum excess work, δΔ*R* = δ*R* – δ*R*^*s*^ – δ*R*^*g*^, i.e., the difference in *R* between the system and the gas/vapor (*g*) and solid (*s*) reference systems. We introduce the following postulates:

A. The effect of an interface is confined within a finite distance, denoted by *v* (volume of the interface), *n*_2_ (number of sorbates within *v*), and *n*_1_ (number of sorbents affecting *n*_2_)
B. Reference systems contribute negligibly to sorbates for vapor sorption with limited absorption into sorbents, so that the surface excess, Δ*n*_2_ = *n*_2_ – *n*_2_^*s*^ – *n*_2_^*g*^, can be approximated as Δ*n*_2_ ≃ *n*_2_.

Using postulates (A) and (B), δΔ*R* can be expressed in a simple manner (see Supporting Information section A) as
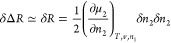
2Noting that δΔ*R*, a positive definite, has an intensive order of magnitude (δΔ*R* = O(1)), it follows that
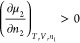
3aas the stability condition for an interface. Assuming the Gaussian distribution, we obtain
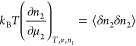
3bwhere *k*_B_ is the Boltzmann constant and ⟨ ⟩ denotes an ensemble average. Note that  and  when the stability condition is satisfied.^47^Equation 3b can be rewritten as
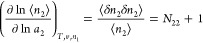
3cwhich is the fundamental equation of the fluctuation sorption theory, i.e., the excess number relationship, which links the ln–ln gradient of an isotherm  to the excess number of sorbates around a probe sorbate, *N*_22_.^5,6,8,52^Equations 3b and 3c have been derived previously via the standard Gibbsian approach (by the μ_2_ derivative of ⟨*n*_2_⟩).^5,6^ However, the relationship between number fluctuation and thermodynamic stability was not clear in this approach. Consequently, the present rederivation via the minimum excess work will be demonstrated to be advantageous for providing a direct connection to thermodynamic stability in nanoscale systems.

#### Nanoscale Stability

Now, we generalize eq 3b to nanoscale systems, such as pores. To achieve this, the macroscopic stability theory (eqs 1–3c) alone is insufficient. The generalization can be executed by employing the following postulates:^47^

C. An ensemble consisting of a macroscopic number  of nanoscale systems obeys the classical macroscopic thermodynamics.^48−50^
D. A stability condition must be written down in an ensemble size-independent manner, per an extensive quantity that characterizes the constituent nanoscale system.^47,53^

Following postulate (C), here, we derive a nanoscale counterpart to eq 3b. The first step is to regard eq 3b as a relationship for a macroscopic ensemble consisting of  nanoscale systems (such as pores). Note that  has the macroscopic order of magnitude. We introduce the following thermodynamic quantities for the nanoscale system denoted with a tilde:
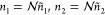
4a

Considering the statistical independence of the nanoscale systems within the macroscopic ensemble,^54^ we obtain the following scaling relationship:
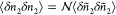
4bUsing eqs 4a and 4b, eq 2 can be rewritten as
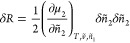
5aAssuming the Gaussian distribution,^47,53,54^ we obtain
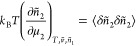
5beq 5b is identical in form to the macroscopic relationship (eq 3b). Thus, we have shown that the sorbate number fluctuation for a nanoscale system can be calculated in a manner analogous to its macroscopic ensemble.

Following postulate (D), we express the quadratic form (eq 5a) and the fluctuation relationship (eq 5b) in a size-invariant manner, using the quantity whose magnitude is characteristic of the nanoscale system. Our previous paper on confined fluids has chosen *ñ*_1_ (i.e., the number of boundary objects) as the characteristic quantity.^47^ Here, instead, we choose ⟨*ñ*_2_⟩ because it is experimentally observable. Under this choice, eqs 5a and 5b become:
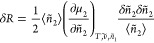
6a
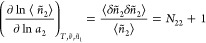
6bNote that *N*_22_ in eq 6b can be rationalized by eq 4b and the constancy of  when carrying out differentiation.

#### Excess Number Relationship from Macroscopic and Nanoscopic Perspectives

We have arrived at an important result: the same excess number *N*_22_ results from the macroscale (eq 3c) and the nanoscale (eq 6b) isotherm gradients, namely
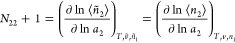
7which guarantees that the isotherm measurement, that is macroscopic in nature, is sufficient to probe the sorbate distribution at the nanoscale. This is the consequence of subdivision into nanoscopic subsystems (postulate (C) and its corollary (eq 4a)). This important relationship (eq 7, referred to as the excess number relationship^9^) relies on the independence of  on *a*_2_. (We will later show how  may differ between the adsorption and desorption branches; see the Results and Discussion section.) The ln–ln gradient of an isotherm, via eq 7, reflects the stability condition both on macroscopic and nanoscopic scales for an interface subdivided into nanoscopic subsystems. (Such a ln–ln gradient may be obtained via a direct numerical differentiation of the raw data or using the fitting functions with physical or empirical origin.)

#### Subdivision Caps Fluctuation and Isotherm Gradient

**Figure 2:**
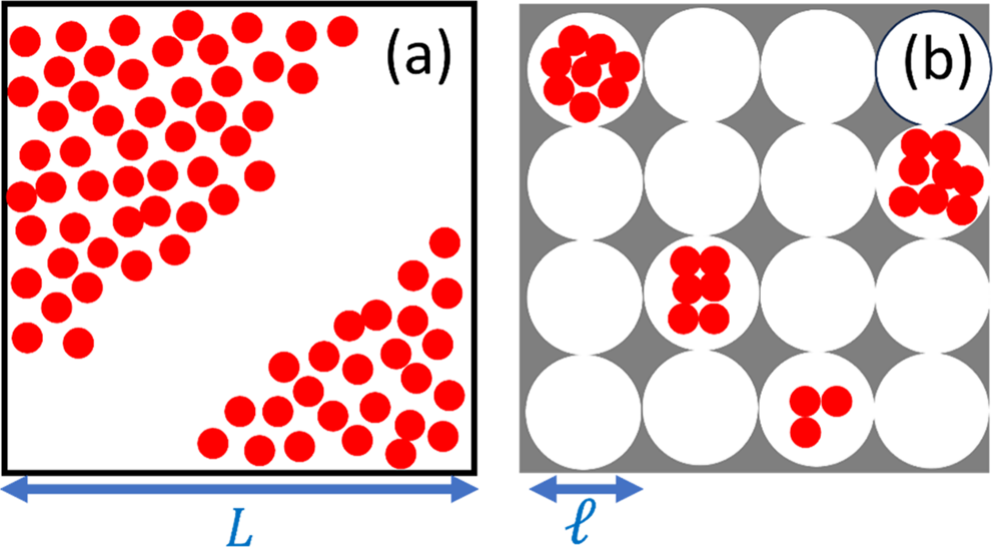
Schematic representation of (a) a macroscopic interface without subdivision viewed from the direction normal to the interface with *L* being its characteristic length scale and (b) a macroscopic interface (represented by the square) subdivided into nanoscopic pores (represented by the circles) with  being their characteristic length scale. The red spheres represent sorbate molecules. Note that  (i.e., the total number of nanoscopic pores, within a macroscopic system) is macroscopic in order, unlike this schematic diagram. While sorbate fluctuation of the macroscopic size scale is possible for an unsubdivided macroscopic system as depicted in (a), subdivision into nanoscale pores in (b) restricts the size scale of fluctuations.

Here, we show that the “transition” of an isotherm cannot be discontinuous when an interface (of the macroscopic characteristic length, *L*) is subdivided into nanoscale “subsystems” (such as pores, with the nanoscopic characteristic length, ). The basic idea for the proof is the following. In the absence of subdivision, sorbate number fluctuation can reach the same order of magnitude as the macroscopic interface (Figure 2a). However, the nanoscale subdivision caps sorbate number fluctuation (Figure 2b), which cannot exceed the nanoscale order of magnitude (Supporting Information section B).

**Figure 3:**
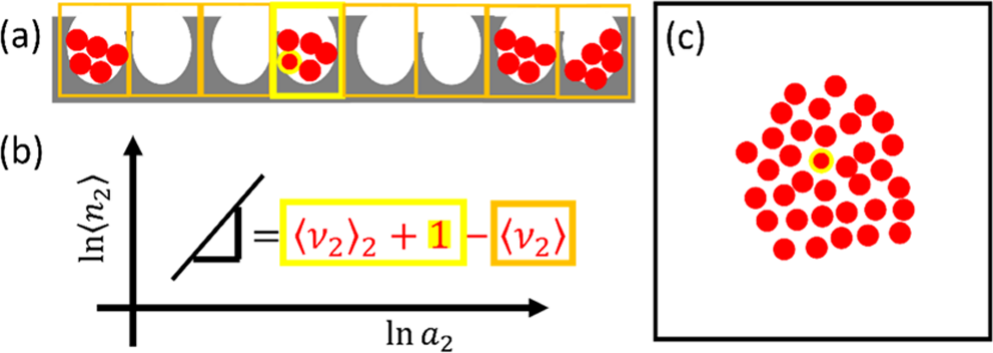
Schematic representation of the derivation of branch isotherm equations based on (a) the subdivision of an interface and enumerating the difference in sorbate number between a pore (highlighted with a yellow box) with a probe sorbate (yellow) and the rest of the pores (with orange boxes) that do not contain the probe. (b) The difference in average sorbate number between the pore with the probe (the probe with ⟨ν_2_⟩_2_ other sorbates, yellow) and the pores without the probe (orange) yields the ln–ln gradient of the isotherm. (c) In the absence of subdivision into nanopores, the number of sorbates around a probe (yellow) may reach a macroscopic order of magnitude, causing a macroscopic-scale deviation from the mean sorbate number in the absence of (away from) the probe. This leads to the divergence of the isotherm’s ln–ln gradient.

First, we show that the sorbate excess number is nanoscopic. To do so, let us express the excess sorbate number in the macroscopic and nanoscopic expressions, which will be the key for demonstrating the nonabruptness of transitions in nanopores. Remembering that *ñ*_2_ and δ*ñ*_2_ are additives under statistical independence (eqs 4a and 4b), substituting eqs 4a and 4b into eq 3c yields
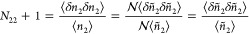
8ashowing that the macroscopic  and nanoscopic  relative fluctuations are the same under subdivision. Consequently, the maximum nanoscopic relative fluctuation,  (Supporting Information section B), is the macroscopic relative fluctuation, as
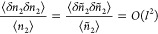
8bSecond, we will show that the isotherm gradient does not diverge to macroscopic scale under the fluctuation cap. To do so, let us combine (i)  is the ln–ln gradient of an isotherm (eq 7 and Figure 3a,b); (ii)  from eq 8b; and (iii) isotherm gradient divergence takes place when , which is intensive (i.e., *O*(1)) under stability conditions, becomes extensive (i.e., *O*(*L*^2^)) or reaches the macroscopic order of magnitude (Figure 3c and Supporting Information section B). However, under subdivision,  is capped at , never reaching *O*(*L*^2^). Hence, the isotherm gradient never diverges, and the isotherm never becomes discontinuous.

From a nanoscopic perspective, fluctuation of the order  violates the stability condition. However, from a macroscopic point of view, such a fluctuation does not break the stability condition. Indeed, there are only intensive (*O*(1)) and extensive (*O*(*L*^2^)) thermodynamic quantities for a macroscale system; hence,  is viewed as intensive  from a macroscopic point of view (Supporting Information section B). Thus, the same fluctuation can be interpreted as (i) liquid–vapor transition (phase instability) in the nanoscale and (ii) local number fluctuation of a stable macroscopic system. These two perspectives will serve as the theoretical foundation for the two alternative approaches (i.e., the fluctuation theory and Hill’s thermodynamics of small systems) to elucidate the interactions underlying isotherm branches in the Results and Discussion section, as well as for proving their equivalence.

### Equations for Adsorption and Desorption Branches

Here, we derive isotherm equations that can describe adsorption and desorption branches (objective II). Our foundation is the sorbate excess number (eq 8a), which has incorporated the effect of subdivision into nanopores. Following our previous paper (with details to be found in eqs 4a–5a of ref (9)), let us consider a simple case, in which sorbate–sorbate correlation is restricted within the same pore. The mean sorbate number, conditional to the presence of the probe within the same cluster, *m* = ⟨ν_2_⟩_2_ + 1, deviates from that of other  clusters, ⟨ν_2_⟩, which do not feel the effect of the probe. Hence, ⟨*n*_2_⟩_2_ of the total interface can be expressed as
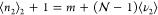
9aIts deviation from the probe-free clusters, , yields
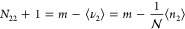
9bby virtue of *N*_22_ = ⟨*n*_2_⟩_2_ – ⟨*n*_2_⟩, which has been represented schematically in Figure 3a,b. With the introduction of fractional saturation
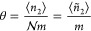
10awe can express the excess number relationship in a compact form
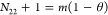
10bwhere *m* is supposed to be constant independent of *a*_2_. Combining eq 10b with eq 7 yields the differential equation for the adsorption and desorption branches, as
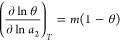
11awhich can be integrated to yield
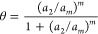
11bwhere *a_m_* is an integration constant that corresponds to the maximum isotherm gradient.^24^ We emphasize here that eq 11b was derived originally as a special case of a more general isotherm that contains adsorption processes involving 1, 2, ···, and ν sorbates sorbing together.^7^ This general isotherm reduces to eq 11b under the condition that the contribution from the sorption of *m* sorbates is dominant.^7^ In doing so, contributions from smaller clusters, which may also be present, have been neglected. However, eq 11b can fit experimental isotherms for porous materials successfully despite its simplicity.^7,24^

As shown in our previous papers, *a*_2_ = *a_m_* is at the steepest isotherm gradient (whose approximate position of *a*_*m*_ can easily be located by the eye). This makes *RT* ln *a*_*m*_, the transfer free energy of a sorbate from the saturated vapor to the interface (see Supporting Information section C), easily accessible from experimental isotherm data. Based on the above, we propose to use the simple cooperative isotherm equation (eq 11b) both for the adsorption and desorption branches. As will be demonstrated in the Results and Discussion section, the sorbate cluster sizes for the adsorption and desorption branches (*m* and *m*′) and their points of steepest gradient (*a_m_* and *a*_*m*′_) will be sufficient to explain the difference between the two branches. Note that eq 11b on its own does not satisfy Henry’s law at *a*_2_ → 0,^7,9,24^ which will be resolved for fitting experimental data (see the Results and Discussion section). Thus, we have derived the isotherm equations for the hysteresis branches (objective III).

The cooperative isotherm (eq 11b), derived quasi-thermodynamically in our previous papers,^7,9,24^ has now been linked to thermodynamic stability condition; the finite gradient of an isotherm comes from finite *m*, arising from the nanoscopic subdivision of a macroscopic interface (Figure 3a). Note that eq 10a assumes simplistically that the cooperative sorption of *m* sorbates fill up the pore. Such a process can capture the salient features of experimental isotherms,^7,9,24^ yet it entirely neglects additional adsorption of sorbates on cooperatively sorbed sorbate clusters. We will later argue that this additional adsorption on clusters plays a key role in the switching from the metastable adsorption branch to desorption (objective III; see the Results and Discussion section).

### Linking an Isotherm Branch to Interfacial Free Energy

To clarify the energetics underlying hysteresis (objective III), we need to evaluate the interfacial free energy underlying an isotherm branch. To do so, the fluctuation sorption theory, from which the branch isotherms have been derived, must be synthesized with the Gibbs Isotherm. (Note that our generalized Gibbs isotherm applies to interfaces with any geometry or porosity, even with sorbent structural changes, because of its ensemble-based foundation.^5,6^ Since the details of the derivation have already been published,^5,6^ we summarize its outlines in Supporting Information section D.) The key quantity is γ_1_, the interfacial free energy per unit amount of sorbent. Using *N*_1_ (the amount of sorbent), the total excess interfacial free energy, *F*_*I*_, can be expressed as (see Supporting Information section D):
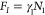
12aFollowing the previous subsection, both *F*_*I*_ and *N*_1_ can be decomposed into nanoscale quantities, *F̃*_*I*_ and *Ñ*_1_, in analogy to eq 4a, via
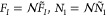
12bCombining eqs 12a and 12b leads to
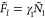
12cThus, γ_1_ signifies the interfacial energy per unit sorbent quantity on the macroscale (eq 12a) as well as on the nanoscale (eq 12c).

With the above preparation, now we show how to evaluate γ_1_ underlying an isotherm. An experimental isotherm is routinely reported as ⟨*n*_2_⟩/*N*_1_, i.e., the amount of sorption per unit sorbent quantity or may be converted to the fractional saturation θ. We start with the generalized Gibbs isotherm (Supporting Information section D)
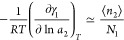
13aIntegrating eq 13a with respect to *a*_2_ yields how γ_1_ changes with *a*_2_
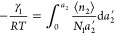
13bwhere *a*_2_^′^ is the variable for integration and γ_1_ = 0 at *a*_2_ = 0 was chosen as its baseline. eq 13b expresses γ_1_ as a function of *a*_2_. An alternative to eq 13b for θ can be obtained by combining eq 13a with eq 10a
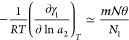
14aHere, we introduce the normalized interfacial free energy
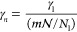
14bexpressed per , the maximum sorption capacity per unit amount of sorbent. Integrating eq 14a, in combination with eq 14b, yields
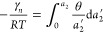
14cThis integration will be executed in the Results and Discussion section to yield the interfacial free energy as a function of sorbate activity.

The excess sorbate cluster number, *N*_22_ + 1, contributes to the gradient of the interfacial free energy. This can be shown by combining eq 7 with the generalized Gibbs isotherm (see Supporting Information section D for derivation), through which we obtain the following result for the macroscopic system:
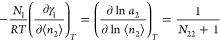
15aThe nanoscale system can be expressed similarly as
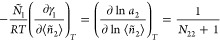
15bNote that the same relationship between *N*_22_ and γ_1_ applies to eq 15a (macroscopic) and 15b (nanoscopic). *N*_22_ + 1 in eqs 15a is a ⟨*n*_2_⟩-gradient (or θ-gradient) of the interfacial free energy, instead of the *a*_2_-gradient in eq 13a. This distinction will play a crucial role when understanding hysteresis via the underlying interfacial free energies; we emphasize that *a*_2_, not ⟨*n*_2_⟩, is the natural variable for isotherms.

To summarize, we have shown how the interfacial free energy can be expressed as a function of sorbate activity (eqs 13b and 14c). Its application to the isotherm equations will reveal the energetic basis of isotherm hysteresis in the Results and Discussion section (objective III).

## Results and Discussion

### Why Transitions Are Sharp yet Continuous (Objective I)

In the Theory section, we have shown that the subdivision of a macroscopic interface into nanoscale subsystems caps the magnitude of fluctuation (Figure 2b). Consequently, the ln–ln gradient of the adsorption and desorption branches (Figure 3a,b) remain finite, thereby achieving objective I.

#### Two Approaches to Hysteresis

The resolution of objective I, via the macroscopic and nanoscopic representations of *N*_22_ + 1 (eq 7), offers two approaches to sorption hysteresis. First, from a macroscopic perspective, a sorbate number fluctuation is still within thermodynamic stability, leading to the derivation of the cooperative isotherm (eq 13b) by the fluctuation sorption theory. Second, from a nanoscopic perspective, the fluctuation breaks the nanoscale stability condition and liquid–vapor phase transition. Consequently, the macroscopic interface (as a whole), when viewed nanoscopically, is no longer homogeneous, necessitating the incorporation of the configurational entropy of arranging liquid and vapor pores throughout the interface. This will lead to a nanoscopic derivation eq 7 as will be demonstrated later.

#### Advantages over Capillary Condensation Models

The difference in gradient between the adsorption and desorption branches is central to the IUPAC hysteresis classification (Figure 1). However, the capillary condensation model focused exclusively on critical activities while neglecting the gradient. In the EOS-based approaches to sorption hysteresis, assuming abrupt transitions has made it impossible to draw any conclusions on sorbate cluster size. In contrast, the relationship between the gradient of an isotherm branch and the sorbate cluster size (eq 8a), derived via the fluctuation theory, is capable of attributing cooperative sorbate cluster size underlying the gradient of isotherm branches.

### Capturing Hysteresis Loop by the Key Mechanistic Parameters (Objective II)

Here, we demonstrate that (i) our cooperative isotherm can be applied to fit the experimental hysteresis loop and (ii) hysteresis types (Figure 1) can be captured via *m* and *a*_*m*_ for adsorption and *m*′ and *a*_*m*′_ for desorption.

#### Application to Ordered Mesoporous Materials (Step (i))

To apply the cooperative isotherm (eq 11b) to experimental data, we have to account for the low *a*_2_ contributions. This can be achieved by the patchwise-additivity principle^24^ by introducing the ABC isotherm for the simpler (nonporous and microporous^52^) surface patches alongside the cooperative isotherm. The following equation can be used both for the adsorption and desorption branches:
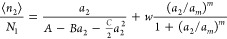
16a

The parameters in eq 16a have a clear physical meaning: *A*, *B*, and *C* signify sorbate-surface, disorbate, and trisorbate interactions, respectively; *w* is the maximum sorption capacity of the cooperative term; *m* is the sorbate cluster number; and *a*_*m*_ is the activity at the steepest isotherm gradient.^6,8,52^ (Note that we have expressed the experimental isotherm explicitly via ⟨*n*_2_⟩/*N*_1_, i.e., the amount of sorption per unit mass of sorbent in eq 16a. When working solely with the excess number relationship eq 8a, as in our previous papers, ⟨*n*_2_⟩/*N*_1_ can be handled as ⟨*n*_2_⟩. However, handling experimental isotherms explicitly as ⟨*n*_2_⟩/*N*_1_ will facilitate the discussion involving γ_1_.)

**Figure 4:**
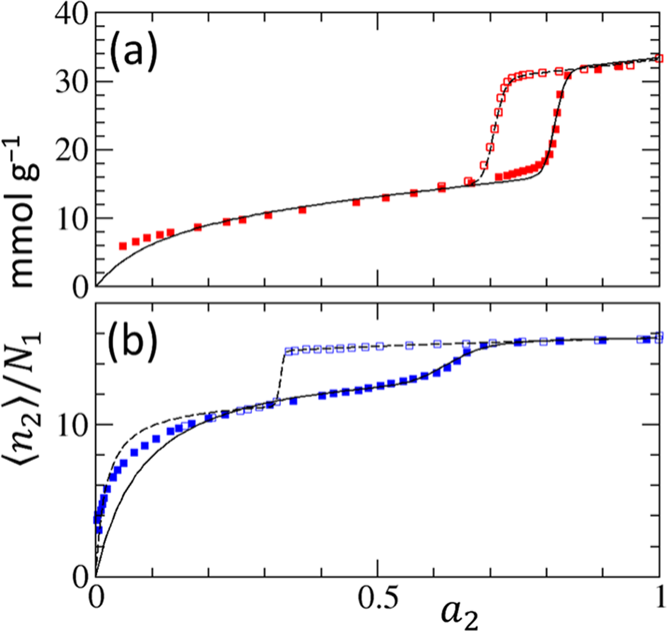
(a) Adsorption of argon on SBA-15. The literature experimental adsorption (red, filled) and desorption (red, open) branches, reported by Villarroel-Rocha et al.,^55^ were fitted with eq 16a (adsorption: solid curve, desorption: dashed curve), with the parameters summarized in Table 1. (b) Adsorption of argon on SBA-16. The literature experimental adsorption (blue, filled) and desorption (blue, open) branches, reported by Villarroel-Rocha et al.,^55^ were fitted with eq 16a (adsorption: solid curve, desorption: dashed curve), with the parameters summarized in Table 1.

The parameters (*A, B, C, w, m, a*_*m*_) can be determined by fitting eq 16a to the isotherm data for both branches. We have chosen the published experimental argon adsorption data on two ordered mesoporous materials, SBA-15 and SBA-16, that exhibit type H1 and type H2 behaviors, respectively.^55^ For SBA-15, eq 16a gives a good fit except for the lowest activity range (Figure 4a and Table 1). For SBA-16, eq 16a could fit the hysteresis region upward, yet this equation showed deviations from experimental data at low *a*_2_, even with the adoption of different (*A*′,*B*′,*C*′) for the desorption branch (Figure 4b and Table 1). However, a *local* fitting of an isotherm, around the region of interest, has been shown to be sufficient for identifying the mechanism around this region.^52^

**Table 1:** Fitting Parameters for Figure 4 (eqs 16a) for the Adsorption and Desorption Branches of Argon on Ordered Mesoporous Materials

	SBA-15^a^		SBA-16^a^	
	adsorption	desorption^b^	adsorption	desorption^b^
*A*	9.75 × 10^–3^	9.75 × 10^–3^	5.90 × 10^–3^	1.52 × 10^–3^
*B*	–6.68 × 10^–2^	–6.68 × 10^–2^	–6.72 × 10^–2^	–8.62 × 10^–2^
*C*	4.12 × 10^–2^	4.12 × 10^–2^	–6.24 × 10^–3^	9.86 × 10^–3^
*w*	1.56 × 10^1^	1.53 × 10^1^	2.55 × 10°	3.62 × 10°
*a_m_*	0.815	0.707	0.625	0.328
*m*	84.9	73.4	18.8	107

a Sample B of Villarroel et al.^55^

b Referring to *A*′, *B*′, *C*, *w*′, *a*_*m′*_, and *m*′ for the desorption branch.

Consequently, mechanistic insights on hysteresis can be drawn from a reasonable regional fitting around the loop.^52^ The difference between the branches, i.e., hysteresis loop, can be attributed to the cooperative parameters, *a*_*m*_, *a*_*m*′_, *m*, and *m*′. Note that *a*_*m*_ (and *a*_*m*′_) and *m* (and *m*′) have a direct relationship to the shape of a branch (Supporting Information section C). Indeed, isotherm fitting shows that (i) reduction from *a*_*m*_ to *a*_*m*′_, signifying the lowering of sorbate free energy at the interface, happens for both SBA-15 and SBA-16; (ii) for SBA-15, a slight reduction in sorbate cluster number from *m* to *m*′ takes place (Table 1); and (iii) for SBA-16, a significant increase of sorbate cluster number from *m* to *m*′ takes place (Table 1).^4^

It is well-known that SBA-15 has a hexagonal array of mesopores with “sponge-like” microporosity on the pore walls.^56^ The functional shape of isotherm branches at lower *a*_2_ has been attributed to micropore filling.^52^ Recently, we have demonstrated that the adsorption on microporous materials can be captured by the ABC isotherm,^52^ which has been adopted in this paper as the first term of eq 16a. Consequently, the capacity of the ABC term to capture the shape of isotherm branches at lower *a*_2_ fulfills its previous attribution to micropore filling.^52^ Intuitively speaking, because of the small number of sorbates involved in micropore filling, taking up to trisorbate interaction is sufficient to capture the micropore filling.

Thus, our theory can capture adsorption on porous materials that involve multiple pore size scales. The ABC term captures micropore filling while mesopore filling is modeled by the cooperative term. We emphasize that our theory does not start with a particular pore geometry (shape, size, or distribution) to construct an isotherm model from bottom up. Instead, our theory focuses on sorbate–sorbent and sorbate–sorbate interactions that are influenced by the geometry of the pores that surround the sorbates. With this alternative approach, not only does our theory provide a simple description of isotherms with a minimum number of assumptions but also reveal the underlying physical mechanism through its parameters.

#### Mechanisms Underlying Hysteresis Types (Step (ii))

Our next step is to capture the IUPAC hysteresis types^4^ via the cooperative isotherm. Let us start with the following simple isotherm equation applicable to both adsorption and desorption branches:
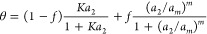
16bvia the weighted (*f*) average of (1) the Langmuir-type isotherm (i.e., a special case of the statistical thermodynamic AB isotherm^8^) with the vapor/interface partition coefficient *K* (as the generalization of the Langmuir constant^8^) and (2) the cooperative isotherm (eq 11b with different parameters for the adsorption and desorption branches). The advantage of eq 16b is in its straightforwardness for integration to obtain the interfacial free energy, compared to a complex form arising from the first term of eq 16a.

**Figure 5:**
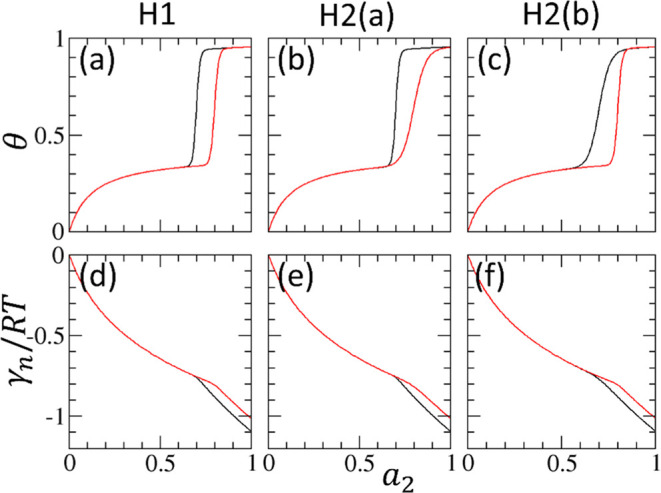
(a) IUPAC type H1 hysteresis reproduced via eq 16b with the parameters *m* = *m*′ = 75. (b) IUPAC type H2(a) hysteresis reproduced with the parameters *m* = 25, *m*′ = 75. (c) IUPAC type H2(b) hysteresis reproduced with the parameters *m* = 75, *m*′ = 25. (The rest of the parameters, *a*_*m*_ = 0.8, *a*_*m*′_ = 0.7, *K* = 8 and *f* = 0.6, were common to panels (a–c).) (d–f) The normalized interfacial free energies, γ_*n*_, underlying the corresponding isotherms (a–c), calculated using eq 17.

While the isotherm behavior at low *a*_2_ comes from the Langmuir-like term, which is common to the adsorption and desorption branches, hysteresis comes predominantly from the cooperative isotherm (the second term of eq 16b; Figure 5a–c).

Type H1 hysteresis shape is observed (Figure 5a) when the sorbate cluster size (*m* = *m*′) and the total number of clusters (nanoscale subsystems) () change little from adsorption to desorption. This is consistent with the IUPAC report: “[u]sually, network effects are minimal”^4^ for type H1.

Type H2(a) hysteresis shape is observed (Figure 5b) when the sorbate cluster size increases (*m*′ > *m*) and the total number of clusters decreases () from adsorption to desorption. This is consistent with some of the common proposals on the mechanism underlying type H2(a). (i) The “network effect” of the pores^4^ is consistent with larger sorbate clusters and a smaller total number of clusters in desorption. (ii) The cavitation-induced evaporation for desorption (i.e., “the bottle could empty via a cavitation process with the neck remaining filled”^57^), caused by “spontaneous local density fluctuation,”^58^ is equivalent to a large *N*_22_, leading to a large *m*′.

Type H2(b) hysteresis shape is observed (Figure 5c) when the sorbate cluster size decreases (*m*′ < *m*) and the total number of clusters increases () from adsorption to desorption. This is consistent with the previous mechanistic proposals. First, “the absence of percolation”^12^ in the IUPAC report is consistent with cluster size decrease (*m*′ < *m*). Second, “pore blocking affected desorption”^59^ (from which “the pore neck size distribution can be calculated from the desorption branch”^4^), if it causes increased subdivision , leads to a smaller sorbate cluster to be desorbed cooperatively (*m*′ < *m*).

Thus, the cooperative isotherm can fit experimental hysteresis data and reproduce types H1, H2(a), and H2(b) of the IUPAC hysteresis classifications. The hysteresis types are distinguished by a comparison of sorbate cluster sizes between the adsorption and desorption branches. However, although the comparison of *m* versus *m*′ governs the *isotherm* hysteresis types, they cannot explain the energetics of hysteresis.

### Energetics of Hysteresis (Objective III)

#### Interfacial Free Energies of the Adsorption and Desorption Branches

Here, we reveal the energetic basis of the hysteresis loop (objective III). To do so, we need to establish a link between an isotherm branch and the interfacial free energy by combining eq 16b with eq 14c, which yields
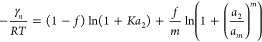
17The application of eq 17 shows that the desorption branch is more stable (in terms of the normalized interfacial free energies) than the adsorption branch (Figure 5d–f).

**Figure 6:**
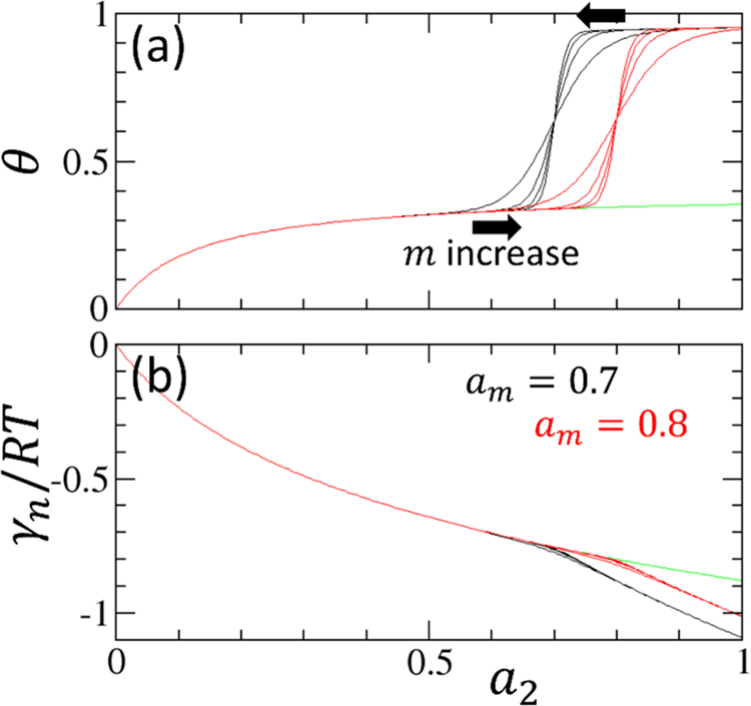
(a) Changing *m* within the same *a*_*m*_ affects the isotherm shape. *f* = 0 (green), *f* = 0.6 and *a*_*m*_ = 0.8 (red). and *f* = 0.6 and *a*_*m*_ = 0.7 (black) at *m* = 20, 40, 60, and 80 (sharing the parameter range for Figure 4 and 5 and Table 1), plotted using eq 16b. (b) Changing *m* within the same *a*_*m*_ hardly affects the sorbate activity dependence of the normalized interfacial free energy (γ_*n*_/*RT*), plotted using eq 17. Unlike the isotherm (a), the cooperative contribution to the interfacial free energy is minor, and the variation of *m* hardly changes γ_*n*_/*RT* for *m* ≥ 20 and shows a limiting behavior.

We have shown above that the IUPAC hysteresis classification is founded on a comparison between the sorbate cluster size between the adsorption and desorption branches. Indeed, types H1, H2(a), and H2(b) *isotherm* loops are all different from one another (Figure 5a–c). However, hysteresis loops, observed via the interfacial free energy, all look very similar (Figure 5d–f). Indeed, unlike θ (Figure 6a), how γ_*n*_ depends on sorbate activity is almost independent of *m* for *m* > 20 (i.e., the parameter range corresponding to ordered mesoporous materials, Table 1), as observed in Figure 6b. Note that when *a*_2_ > *a_m_*, the second term of eq 17, at *m* → ∞, tends to an *m*-independent form, .

However, there is a clear gap in the interfacial free energies between the adsorption and desorption branches (Figure 6b). Since this gap is regardless of *m*, it is driven by *a*_*m′*_ < *a*_*m*_ or increased stability of a sorbate molecule for the desorption branch, as inferred from the transfer free energy of a sorbate (from saturated vapor to the interface, *RT* ln *a*_*m*′_ < *RT* ln *a*_*m*_).

To summarize, the difference in sorbate cluster size between the adsorption and desorption branches, while playing the major role in classifying the hysteresis loops based on isotherms, is a minor contribution to the interfacial free energy difference within a branch (Figure 6). While *N*_22_ + 1, hence *m*, is the gradient (first-order derivative) of isotherm (eq 8a), it is also a second-order derivative of the interfacial free energy (via eqs 8a and 13a). Consequently, *m* is useful for isotherm classification but plays a secondary role in interfacial free energies.

#### Switching from Adsorption to Desorption (Step (iii))

The interfacial free energy underlying the cooperative isotherm has clarified that (1) the stabilization of the desorption branch (i.e., lowering of the interfacial free energy, γ_*n*_) is the key to the emergence of sorption hysteresis and that (2) stabilization comes predominantly from the increase in sorbate stability of the desorption branch (*a*_*m*′_ < *a*_*m*_; or a more negative sorbate transfer free energy from the saturated vapor to the interface, *RT* ln *a*_*m*′_ < *RT* ln *a*_*m*_). Here, we discuss its possible mechanisms. First, percolation/network effects, proposed for type H2(a),^4^ can strengthen the sorbate–sorbate and sorbate–interface interactions, leading to increased sorbate stability. A sufficient chance of pores neighboring, with additional sorption for connecting the pore, is its possible mechanism. We emphasize that it is *a*_*m*′_ < *a*_*m*_, not *m*′ > *m*, that is the key to hysteresis. Second, pore blocking (“on desorption the bottle cannot empty until the necks are emptied”^57^) has been inferred for both types H2(a) and H2(b),^4^ despite their opposite behavior in the change of sorbate cluster sizes (*m*′ > *m* and *m*′ < *m*). However, as in the case of percolation, the difference between *m* and *m*′ (despite its dominant role in the isotherm shape) plays a minor role in the interfacial free energy. Consequently, “pore blocking” should be chiefly about sorbate stabilization induced by additional postcooperative sorption that maximizes sorbate–interface contact. Note that sorbent structure changes that our theory can incorporate may also contribute to the above stabilization. Thus, *a*_*m*′_ < *a*_*m*_ plays a dominant energetic role, whereas *m*′ and *m* play a minor role, which is crucial for translating the proposed hysteresis mechanisms to a language of energetics and stability.

### Branch Isotherm Equations from Hill’s Thermodynamics of Small Systems (Objective IV)

The statistical thermodynamic fluctuation theory led to (i) the cooperative isotherms for adsorption and desorption branches in the Theory section, (ii) the interfacial free energy representation of the hysteresis loop, and (iii) a link between (i) and (ii) to the mechanisms of switchover (i.e., sorbate stabilization and sorbate cluster growth via percolation) in the previous subsection. Objective III was already addressed *macroscopically*, enabling us to elucidate a hysteresis loop based on a few parameters that capture its mechanism.

#### Perspective from Hill’s Thermodynamics of Small Systems

The goal of this subsection is to demonstrate that the branch isotherm eq (eq 11b) can also be derived from Hill’s thermodynamics of small systems.^48−50^ From the perspective of small systems, thermodynamic instability induces vapor/liquid transition, making each of the nanopores take one of the two states (i.e., *A*: filled, *B*: unfilled). Our goal is to express the interfacial free energy *F*_*I*_ in terms of the numbers ( and ) and the interfacial energies (γ_*A*_ and γ_*B*_) of each pore state. (Here, we summarize the outline, leaving the details of derivation to Supporting Information section E.) We assume the following form for *F*_*I*_:^49^
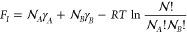
18where the final term is the entropy of arranging two pore types. In the absence of sorbates, , *F*_*I*_ = 0 must hold; hence, γ_*B*_ = 0, which signifies the zero interfacial free energy contribution from an empty pore. (The discussion here is analogous to Hill’s two-state model for phase transitions in small systems on p119 of ref (49)). Minimizing *F*_*I*_ with respect to  (while keeping , the number of total pores, constant) under Stirling’s approximation, we obtain
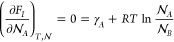
19Combining eqs 18 and 19 (under Stirling’s approximation, see Supporting Information section E), yields
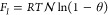
20Rewriting eq 20 using the definition of γ_1_ (eq 12a) and differentiating it with respect to θ leads to
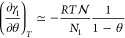
21aby restoring ⟨*n*_2_⟩ via eq 10a, eq 21a becomes
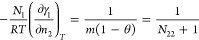
21bwhich leads via eq 15a to *N*_22_ + 1 = *m*(1 – θ), identical to eq 10b from the fluctuation theory. Thus, the cooperative isotherm for sorption branches (eq 11b) was also derived from Hill’s thermodynamics of small systems (objective III).

#### Nanopore as a Pseudophase

When working with nanoscopic systems, it is important to clarify how they relate to experimental measurements. Experimental isotherms are macroscopic, defined as the surface excess of sorbates (i.e., approximated as ⟨*n*_2_⟩/*N*_1_ for sufficiently strong sorption detectable by measurements). According to the Gibbs phase rule, a two-component system (sorbate and sorbent) forming two phases (sorbate vapor and sorbent) has *F* = 2–2 + 2 = 2 degrees of freedom. Under constant temperature, one degree of freedom is left for sorbate activity (or relative pressure) without any room for additional phase equilibria. We emphasize that complexity in interfacial geometry (e.g., porous), or the consequent number fluctuation, does not add an extra degree of freedom.

From a perspective of macroscopic thermodynamics, we are dealing with inhomogeneity in sorbate distribution that can be captured by *N*_22_.^22,60^ Consequently, the nanoscale “phase” transition (or “condensation” and “evaporation” in the context of the capillary condensation model) does not refer to real phases, as has been made clear by the Gibbs phase rule. We emphasize here that the arrangement of filled and unfilled pores at a value of sorbate activity (whose entropy is the foundation for deriving the cooperative isotherm from the small systems perspective, via eq 21b) means that the “phase boundary” is blurred, which is true especially for small *m* or *N*_22_ + 1. Instead, nanophases can be considered as pseudophases convenient for studying finite-sized clusters, just like the treatment of micelles as a phase (Supporting Information section F).^22,60^

We emphasize also that our statistical thermodynamic fluctuation theory does not introduce any explicit assumptions on the “state” of the pseudophase. Consequently, our theory is valid for liquid-like and “solidified pore condensates”^61^ alike and hence can deal with capillary sublimation as has been reported for argon at low temperatures.^61^

#### Comparison of the Two Perspectives

First, we compare the fluctuation theory and Hill’s thermodynamics of small systems^48−50^ as applied to the sorption onto porous interfaces:

- The fluctuation theory is *local* in its perspective. It focuses on sorbate–sorbate number correlation or, equivalently, the excess number of sorbates around a probe sorbate.
- Hill’s thermodynamics of small systems^48−50^ is *global* in its perspective. It focuses on the entropy of distributing phase-separated pores throughout the system.

For the simple case (i.e., an interface constituting statistically uncorrelated pores), the two perspectives lead to mathematically equivalent branch isotherm equations, in which Hill’s thermodynamics of small systems^48−50^ captures fluctuation via the arrangement of filled/empty pores. Despite the demonstrated equivalence between the two, we point out that the fluctuation theory shares its common language (i.e., molecular distribution functions as the foundation of number correlations) with molecular simulations and liquid theory. Hill’s thermodynamics of small systems,^48−50^ despite its appeal arising from the use of elementary thermodynamic concepts like mixing entropy, makes an indirect link to molecular distribution functions.

## Conclusions

This paper has aimed to answer the following fundamental questions on sorption hysteresis:

I. Why are the transitions sharp yet continuous (see Figure 1)?
II. What is the energetic basis of sorption hysteresis?
III. How can we derive an isotherm equation for hysteresis loops with its parameters with direct mechanistic relevance?
IV. What is the relationship between the fluctuation theory and Hill’s thermodynamics of small systems?

These questions are key to linking the current mechanistic insights underlying the IUPAC hysteresis classification to isotherm equations to the underlying thermodynamic principles. To answer these questions, a statistical thermodynamic foundation is necessary. To this end, this paper has

1. established the thermodynamic stability condition for the macroscale and for the constituent nanoscale;
2. derived the branch isotherm eq (Figure 1, bottom) and identified the sorbate cluster size and per-sorbate stability as its key parameters;
3. expressed how the interfacial free energy for hysteresis branches changes with the sorbate activity; and
4. established how fluctuation can be viewed alternatively via the arrangement of vapor and liquid pores.

These new theoretical tools enabled us to investigate hysteresis via not only isotherm shapes but also underlying energetics.

The transitions are continuous because of the nanoscale subdivision of an interface, which caps the fluctuation scale (question I). Even when sorbate excess number (*N*_22_) breaks nanoscale stability, the macroscopic interface (constituting a macroscopic number of nanoscale systems) is well within the thermodynamic stability.

Isotherm equations for hysteresis branches were derived from two perspectives, macroscopically from the statistical thermodynamic fluctuation theory (question II) and Hill’s thermodynamics of small systems^48−50^ (question IV). The capacity for two equivalent approaches has been demonstrated in the case of cooperative sorption when multiple sorbates sorb together as a cluster. While the derivation from the fluctuation theory focused on an enumeration of sorbate excess numbers, the thermodynamics of small systems focused on the entropy of distributing filled and unfilled pores.

A clear picture of hysteresis has been presented via the interfacial free energies underlying hysteresis branches (question III). The key points are (i) the lower interfacial free energy of the desorption branch and (ii) a switch-over from the adsorption branch to desorption. The driving force for (i) was identified as the increased sorbate stabilization for the desorption branch (*a*_*m*′_ < *a*_*m*_). Even though the difference in sorbate cluster size between adsorption and desorption branches (*m* and *m*′) plays a major role in determining hysteresis shapes underlying the IUPAC classifications, the energetics of hysteresis come predominantly from sorbate stabilization (*a*_*m'*_ < *a*_*m*_). Consequently, the proposed mechanisms of hysteresis, such as “pore blocking”, “percolation”, and “cavitation,” should be chiefly about sorbate stabilization. Indeed, while *m* and *m*′ are the gradients (first-order derivatives) of isotherm branches, thereby an important feature of isotherm hysteresis, they are the second-order derivatives of interfacial free energies; hence, their role is minor in the energetics of hysteresis.

The generality of the fluctuation sorption theory offers advantages over the previous thermodynamic approaches. The sorbate excess number provides a direct microscopic insight into the structure and distribution of sorbate molecules at the interface. Such a *local* insight is inevitably replaced with the *global* distribution of filled pores according to Hill’s thermodynamics of small systems.^48−50^ However, the local view is closer to molecular simulations through the direct relationship between excess energy and molecular distribution functions. Thus, the universal facility of the fluctuation theory in capturing inhomogeneity in sorbate distribution at the interface has been demonstrated through the elucidation of sorption hysteresis. Application to scanning isotherm loops^62^ will be presented in a forthcoming paper.

## Supporting Information Available

Quasi-thermodynamic stability theory; macroscopic versus nanoscopic stability conditions; relating the cooperative isotherm parameters with the functional shape; generalized Gibbs isotherm; nanothermodynamic rederivation; nanophase as the pseudophase (PDF)
