# Supplementary material for: Sorption Hysteresis: A Statistical Thermodynamic Fluctuation Theory
Source: Langmuir. 2024 May 23;40(22):11504–15. doi: 10.1021/acs.langmuir.4c00606 (PMC11155257; doi:10.1021/acs.langmuir.4c00606)
Supplement: Supplementary file 1 — la4c00606_si_001.pdf [file la4c00606_si_001.pdf]

Supporting information

Sorption Hysteresis: A Statistical Thermodynamic Fluctuation Theory

Seishi Shimizu[1,*] and Nobuyuki Matubayasi[2]
[1]York Structural Biology Laboratory, Department of Chemistry, University of York, Heslington, York YO10 5DD, United Kingdom.
[2]Division of Chemical Engineering, Graduate School of Engineering Science, Osaka University, Toyonaka, Osaka 560-8531, Japan
Corresponding Author: Seishi Shimizu: York Structural Biology Laboratory, Department of Chemistry, University of York, Heslington, York YO10 5DD, United Kingdom.
Email: seishi.shimizu@york.ac.uk

Table of Contents

A. Quasi-thermodynamic stability theory. p.S1.
B. Macroscopic versus nanoscopic stability conditions. p.S2.
C. Relating the cooperative isotherm parameters with the functional shape. p.S3.
D. The generalized Gibbs isotherm. p.S3.
E. A nanothermodynamic rederivation. p.S5.
F. Nanophase as pseudophase. p.S5.

A. Quasi-thermodynamic stability theory

Quasi-thermodynamic theory is advantageous over the Gibbsian ensemble approach for establishing a clear link between sorbate number fluctuation to thermodynamic stability. We consider a system surrounded by the reservoir. The key quantity is the minimum excess work done by an external medium on the system + reservoir, δR, that accompanies the exchange of sorbates (species 2)

$$\delta R = \delta E + \delta E^{(r)} \tag{A1}$$

where δE is the change in the energy of the system and $\delta E^{(r)}$ is the change of energy of the reservoir. The superscript (r) represents the reservoir. $\delta E^{(r)}$ accompanying the exchange of matter can be expressed as[1]

$$\delta E^{(r)} = T^{(r)}\delta S^{(r)} + \mu_2^{(r)}\delta N_2^{(r)} \tag{A2}$$

Since the system and the reservoir are in equilibrium, $\mu_2^{(r)} = \mu_2$ and $T^{(r)} = T$ hold. Hence, we use μ_2 and T without the superscript. The conservation of particles and energy between the system and the reservoir leads to $\delta N_2^{(r)} = -\delta N_2$ and $\delta S^{(r)} = -\delta S$. Taken all together, eqs A1 and A2 can be rewritten as

$$\delta R = \delta E - T\delta S - \mu_2\delta N_2 = \delta F + S\delta T - \mu_2\delta N_2 \tag{A3}$$

with the Helmholtz free energy, F. Under constant T, expanding F in terms of δN_2 yields

$$\delta F = \left(\frac{\partial F}{\partial N_2}\right)_{T,V,N_1}\delta N_2 + \frac{1}{2}\left(\frac{\partial^2 F}{\partial N_2^2}\right)_{T,V,N_1}\delta N_2\delta N_2 \tag{A4}$$

Combining eqs A3 and A4 yields

$$\delta R = S\delta T + \frac{1}{2}\left(\frac{\partial \mu_2}{\partial N_2}\right)_{T,V,N_1}\delta N_2\delta N_2 \tag{A5}$$

because of $\mu_2 = \left(\frac{\partial F}{\partial N_2}\right)_{T,V,N_1}$.

Now we apply the stability theory to the interface. This can be achieved by repeating the same argument for the system (which contains an interface) and the reference systems on the solid and gas/vapor sides, denoted by s and g. Subtracting R for the reference systems from that of the system, we obtain the following result for the interfacial minimum excess work, $\Delta R \equiv R - R^s - R^g$:

$$\delta\Delta R = \frac{1}{2}\left(\frac{\partial \mu_2}{\partial N_2}\right)_{T,V,N_1}\delta N_2 \delta N_2 - \frac{1}{2}\left(\frac{\partial \mu_2}{\partial N_2^s}\right)_{T,V^s,N_1}\delta N_2^s \delta N_2^s - \frac{1}{2}\left(\frac{\partial \mu_2}{\partial N_2^g}\right)_{T,V^g}\delta N_2^g \delta N_2^g \quad \text{(A6)}$$

Neglecting the second and third terms of eq A6 following the postulates in the main text, we obtain

$$\delta\Delta R \simeq \delta R = \frac{1}{2}\left(\frac{\partial \mu_2}{\partial n_2}\right)_{T,v,n_1}\delta n_2 \delta n_2 \quad \text{(A7)}$$

which will be used as the foundation of thermodynamic stability theory for an interface.

B. Macroscopic versus nanoscopic stability conditions

Despite the same excess number (N_{22}) underlying the ln-ln gradients of macroscale and nanoscale isotherms (eq 7), there is a crucial difference between the two regarding how the stability condition is broken. To demonstrate this, we will carry out the order-of-magnitude analysis with and without nanoscopic subdivision.

Macroscopic System without Subdivision. For macroscopic systems, there are two types of thermodynamic quantities, intensive and extensive.[1,2] When there is no subdivision into nanoscopic subsystems (pores), a macroscopic interface, with its characteristic length-scale L, is two-tiered in terms of its thermodynamic quantities:[3] $O(1)$ (intensive) and $O(L^2)$ (extensive). As shown above, when the stability condition is satisfied, $\frac{\langle \delta n_2 \delta n_2 \rangle}{\langle n_2 \rangle} = O(1)$. Since the system is two-tiered with $O(1)$ and $O(L^2)$, $N_{22} + 1 = \frac{\langle \delta n_2 \delta n_2 \rangle}{\langle n_2 \rangle}$, when the stability condition is broken, must reach $O(L^2)$. This takes place when the standard deviation reaches the system size order, $\sqrt{\langle \delta n_2 \delta n_2 \rangle} = O(L^2)$. Consequently, the ln-ln gradient of the isotherm diverges, or precisely stated, becomes macroscopic ($O(L^2)$) in its order of magnitude. However, as will be shown in the **Results and Discussion**, when a macroscopic interface is subdivided into nanoscopic subsystems, subdivision makes fluctuations in the order of the entire macroscopic system $O(L^2)$ impossible.

Nanoscopic System. Just like macroscopic systems, a nanoscopic system has intensive and extensive quantities. Using its characteristic length-scale ℓ, they are expressed as $O(1)$ and $O(\ell^2)$. However, a nanoscale ℓ is viewed from a macroscopic perspective as $\ell = O(1)$ because ℓ is below $O(L^2)$. Consequently, $O(1)$ and $O(\ell^2)$ from a nanoscopic perspective are both regarded as $O(1)$ from a macroscopic perspective. Another characteristic of the nanoscale system is the presence of physical quantities of $O(\ell)$ (such as the curvature term), making it

three-tiered with $O(1)$, $O(\ell)$, and $O(\ell^2)$.[4] Consequently, the nanoscopic thermodynamic stability, $N_{22}+1=\frac{\langle\delta\tilde{n}_2\delta\tilde{n}_2\rangle}{\langle\tilde{n}_2\rangle}=O(1)$,[3] could be surpassed only with $O(\ell)$. Even when $N_{22}+1$ reaches $O(\ell^2)$, it is still $O(1)$ from a macroscopic perspective. Indeed, $O(1)$, $O(\ell)$, and $O(\ell^2)$ are all regarded as $O(1)$ from a macroscopic perspective. Hence, the ln-ln gradient of a *macroscopic* isotherm does not diverge (i.e., become $O(L^2)$) even when the nanoscale stability condition is broken.

C. Relating the cooperative isotherm parameters with the functional shape

First, we show that a_m and m has a clear relationship to the functional shape of an isotherm, thereby eliminating ambiguity in the nonlinear fitting of experimental isotherm data. As shown in our recent paper, a_m is the activity at the steepest gradient of an isotherm, $hm/4a_m$, where h is the step height (i.e., the difference between the lowest and highest) of the cooperative isotherm (see Appendix A of Ref [5]). Consequently, with a reasonable estimate of h, m and a_m can either be obtained visually from an experimental isotherm or be used to check the validity of the parameters determined via nonlinear fitting.

Second, we establish what the "nearly parallel"[6] adsorption and desorption branches mean in the framework of the cooperative isotherm. Since nearly parallel means nearly equal gradient,

$$\frac{hm}{4a_m}=\frac{hm'}{4a_{m'}} \tag{C1}$$

because the isotherm step is common. Consequently,

$$\frac{m'}{m}=\frac{a_{m'}}{a_m} \tag{C2}$$

Since $a_{m'}$ is lower than a_m, m' is lower than m. In fact, there is a slight reduction from m to m' as observed for the nearly parallel SBA-15 in **Table 1**. However, for a reasonably narrow isotherm loop, $a_{m'}$ is not too different from a_m. In this case, m' and m can be considered comparable.

D. The generalized Gibbs isotherm

Here we demonstrate that our approach can be applied naturally to interfaces of arbitrary geometry and porosity, unlike the conventional Gibbs isotherm. To do so, as in section A, we consider the system that contains an interface and the two reference systems without an interface (gas/vapor and solid, denoted by the superscripts g and s).[7–9] Instead of the conventional derivation of the Gibbs isotherm from the trio of Gibbs-Duhem equations plus the concentration profile,[7–10] we adopt a novel statistical thermodynamic approach based on Legendre transformation.[11] For the conventional approach, a clearly defined coordinate system is indispensable, which introduces an unnecessary restriction to planar interfaces.[11] Our approach is free from such a restriction, applicable to any surface geometry and porosity.[11,12]

We start with the definition of excess free energy, F_I, via

$$F_I=\Omega-\Omega^g-\Omega^s \tag{D1}$$

as the difference in the thermodynamic function ($\Omega=-PV$) between the entire system (without superscript) and the two reference systems (superscripts g and s) under the

conservation of volume.[11,12] The Gibbs dividing surface can be introduced via Legendre transformation, converting the thermodynamic function Ω (open to species 1 and 2) to the thermodynamic function $Y = \Omega + \mu_1 N_1$ (open to species 2 but closed to 1), as

$$F_I = Y - Y^g - Y^s - \mu_1\left(N_1 - N_1^g - N_1^s\right) \quad \text{(D2)}$$

using μ_1, the chemical potential of species 1.[11] The Gibbs dividing surface, in this setup, is simply to impose the condition $N_1 - N_1^g - N_1^s = 0$, which yields

$$F_I = Y - Y^g - Y^s \quad \text{(D3)}$$

Using the partition function for the partially open ensembles, we can relate F_I to the

$$-\frac{1}{RT}\left(\frac{\partial F_I}{\partial \ln a_2}\right)_T = \langle N_2\rangle - \langle N_2^g\rangle - \langle N_2^s\rangle \quad \text{(D4)}$$

Under the postulate that the effect of an interface is confined within a finite distance from the surface,[11] eq D4 can be rewritten while appreciating the finite-distanced nature of the interface, as

$$-\frac{1}{RT}\left(\frac{\partial F_I}{\partial \ln a_2}\right)_T = \langle n_2\rangle - \langle n_2^g\rangle - \langle n_2^s\rangle \quad \text{(D5)}$$

Using postulate (B) of the main text, eq D5 can be expressed in an approximate manner as

$$-\frac{1}{RT}\left(\frac{\partial F_I}{\partial \ln a_2}\right)_T \simeq \langle n_2\rangle \quad \text{(D6)}$$

Introducing the excess energy per nanoscale system via $F_I = \mathcal{N}\tilde{F}_I$, we can rewrite eq D6 as

$$-\frac{1}{RT}\left(\frac{\partial \tilde{F}_I}{\partial \ln a_2}\right)_T \simeq \langle \tilde{n}_2\rangle \quad \text{(D7)}$$

which is the nanoscale version of eq D6.

Combining eq D6 with the interfacial free energy per unit sorbent, γ_I, defined via eq 12a, we obtain

$$-\frac{1}{RT}\left(\frac{\partial \gamma_1}{\partial \ln a_2}\right)_T \simeq \frac{\langle n_2\rangle}{N_1} \quad \text{(D8)}$$

which is the generalization of the Gibbs isotherm. The nanoscale counterpart of eq D8 can be derived by introducing the amount of sorbent per nanoscale system, $\tilde{N}_1$, defined via $N_1 = \mathcal{N}\tilde{N}_1$, which converts eq D8 into

$$-\frac{1}{RT}\left(\frac{\partial \gamma_1}{\partial \ln a_2}\right)_T \simeq \frac{\langle \tilde{n}_2\rangle}{\tilde{N}_1} \quad \text{(D9)}$$

Our goal is to relate γ_1 to N_{22}. When the isotherm is a single-valued function, we can rewrite the macroscale relationship (eq D8) via the chain rule to yield

$$-\frac{1}{RT}\left(\frac{\partial \gamma_1}{\partial \langle n_2\rangle/N_1}\right)_T\left(\frac{\partial \langle n_2\rangle/N_1}{\partial \ln a_2}\right)_T \simeq \frac{\langle n_2\rangle}{N_1} \quad \text{(D10a)}$$

which leads to

$$-\frac{N_1}{RT}\left(\frac{\partial \gamma_1}{\partial \langle n_2\rangle}\right)_T = \left(\frac{\partial \ln a_2}{\partial \ln\langle n_2\rangle}\right)_T = \frac{1}{N_{22}+1} \quad \text{(D10b)}$$

The nanoscale counterpart (eq D9) can be rewritten in a similar manner, via

$$-\frac{1}{RT}\left(\frac{\partial \gamma_1}{\partial \langle \tilde{n}_2\rangle/\tilde{N}_1}\right)_T\left(\frac{\partial \langle \tilde{n}_2\rangle/\tilde{N}_1}{\partial \ln a_2}\right)_T \simeq \frac{\langle \tilde{n}_2\rangle}{\tilde{N}_1} \quad \text{(D11a)}$$

which leads to

$$-\frac{\tilde{N}_1}{RT}\left(\frac{\partial \gamma_1}{\partial\langle\tilde{n}_2\rangle}\right)_T = \left(\frac{\partial \ln a_2}{\partial \ln\langle\tilde{n}_2\rangle}\right)_T = \frac{1}{N_{22}+1} \quad \text{(D11b)}$$

Note that the same excess number, N_{22}, can be derived from eq D11a (macroscopic) and D11b (nanoscopic).

E. A nanothermodynamic rederivation

Here we provide an alternative derivation of the cooperative sorption isotherm by adapting Hill's two-state model for phase transitions in small systems (see p119 of Ref [4]). The interfacial free energy is denoted as F_I. Let the pores take only the two states: A (filled) and B (unfilled), and the corresponding interfacial energies for the pores be γ_A and γ_B. With this, the interfacial free energy may be written as

$$F_I = \mathcal{N}_A\gamma_A + \mathcal{N}_B\gamma_B - RT\ln\frac{\mathcal{N}!}{\mathcal{N}_A!\mathcal{N}_B!} \quad \text{(E1)}$$

where $\mathcal{N}_A$ and $\mathcal{N}_B$ are the number of pores with states A and B. Using Stirling's approximation

$$F_I \simeq \mathcal{N}_A\gamma_A + (1-\mathcal{N}_A)\gamma_B - RT\mathcal{N}\ln\mathcal{N} + RT\mathcal{N}_A\ln\mathcal{N}_A + RT\mathcal{N}_B\ln\mathcal{N}_B \quad \text{(E2)}$$

In the absence of sorbates at the interface, $\mathcal{N}_A = 0$ and $\mathcal{N} = \mathcal{N}_B$. Under this condition, the interfacial free energy, $F_I = 0$. Consequently, $\gamma_B = 0$, signifying that the surface free energy contribution from an empty pore is zero. Now we minimize F_I with respect to $\mathcal{N}_A$, while keeping $\mathcal{N}$ constant. This yields:

$$\left(\frac{\partial F_I}{\partial \mathcal{N}_A}\right)_{T,\mathcal{N}} = 0 = \gamma_A + RT\ln\frac{\mathcal{N}_A}{\mathcal{N}_B} \quad \text{(E3)}$$

Substituting eq E3 into eq E2 and eliminating γ_A, we obtain

$$F_I = RT\mathcal{N}\ln\left(\frac{\mathcal{N}_B}{\mathcal{N}}\right) \quad \text{(E4)}$$

With this preparation, now we can make a connection to the cooperative sorption theory. Since B is the unfilled pore, using the definition of fractional saturation (eq 10a), we obtain

$$\frac{\mathcal{N}_B}{\mathcal{N}} = 1-\theta \quad \text{(E5)}$$

Here we employ the definition of γ_1 (the interfacial free energy per unit quantity of sorbates) given by eq 12a to rewrite eq E4 as

$$\gamma_1 = RT\frac{\mathcal{N}}{N_1}\ln(1-\theta) \quad \text{(E6)}$$

Differentiating eq E6 with respect to θ yields

$$\left(\frac{\partial\gamma_1}{\partial\theta}\right)_T = -\frac{RT\mathcal{N}}{N_1}\frac{1}{1-\theta} \quad \text{(E7)}$$

The equivalence between eq E7 and the fluctuation theory is discussed in the main text.

F. Nanophase as pseudophase

Here we provide a pseudophase interpretation of sorbate excess numbers, expressed via eq 10b. When viewed nanoscopically, a pore takes the two states: filled and unfilled. Following the common practice of pseudophase thermodynamics, we can introduce the phenomenological unfilled/filled equilibrium constant K for a pore, as

$$K = \frac{1-\theta}{\theta} \quad \text{(F1)}$$

through which the excess number (eq 10b) can be expressed as

$$N_{22} + 1 = \frac{mK}{1+K} \tag{F2}$$

In this manner, the excess number (N_{22}) from the fluctuation theory can be interpreted as the unfilled/filled equilibrium constant (K). (This is mathematically similar to a relationship between the solute-surfactant excess number and the micelle/water partition coefficient of a solute (see eq 3.2a of Ref [13]).) Despite its intuitive appeal, the divergence of K at small θ (see eq F1) poses limitations to this approach.

References

(1) Landau, L. D.; Lifshitz, E. M. Statistical Physics, 3rd Edition, Part I; Pergamon Press: London, 1986; pp 111–124.

(2) Prigogine, I.; Defay, R. Thermodynamique Chimique; Desoer: Liege, 1950; pp 70–73.

(3) Shimizu, S.; Matubayasi, N. Phase Stability Condition and Liquid–Liquid Phase Separation under Mesoscale Confinement. *Physica A* **2021**, *563*, 125385. https://doi.org/10.1016/j.physa.2020.125385.

(4) Hill, T. L. *Thermodynamics of Small Systems*; Dover Publications: New York, 1963.

(5) Dalby, O.; Abbott, S.; Matubayasi, N.; Shimizu, S. Cooperative Sorption on Heterogeneous Surfaces. *Langmuir* **2022**, *38* (43), 13084–13092. https://doi.org/10.1021/acs.langmuir.2c01750.

(6) Sing, K. S. W.; Everett, D. H.; Haul, R. A. W.; Moscou, L.; Pierotti, R. A.; Rouquerol, J.; Siemieniewska, T. Reporting Physisorption Data for Gas/Solid Systems with Special Reference to the Determination of Surface Area and Porosity. *Pure Appl. Chem.* **1985**, *57*, 603–619. https://doi.org/10.1351/pac198557040603.

(7) Gibbs, J. W. The Collected Works of J. W. Gibbs; Yale University Press: New Haven, CT, 1928; pp 219–237.

(8) Defay, R.; Prigogine, I. Tension Superficielle et Adsorption; Desoer: Liege, 1966; pp 71–79.

(9) Adamson, A. W.; Gast, A. P. Physical Chemistry of Surfaces; Wiley: New York, 1997; pp 599–684.

(10) Butt, H. H.-J.; Graf, K.; Kappl, M. Physics and Chemistry of Interfaces; Wiley-VCH: Weinheim, 2013; pp 229–265. https://doi.org/10.1002/3527602313.

(11) Shimizu, S.; Matubayasi, N. Fluctuation Adsorption Theory: Quantifying Adsorbate-Adsorbate Interaction and Interfacial Phase Transition from an Isotherm. *Phys. Chem. Chem. Phys.* **2020**, *22*, 28304–28316. https://doi.org/10.1039/D0CP05122E.

(12) Shimizu, S.; Matubayasi, N. A Unified Perspective on Preferential Solvation and Adsorption Based on Inhomogeneous Solvation Theory. *Physica A* **2018**, *492*, 1988–1996. https://doi.org/10.1016/j.physa.2017.11.113.

(13) Shimizu, S.; Matubayasi, N. Cooperativity in Micellar Solubilization. *Phys. Chem. Chem. Phys.* **2021**, *23* (14), 8705–8716. https://doi.org/10.1039/d0cp06479c.
